# Tissue-specific biosynthesis and regulation of alkaloids, flavonoids, and terpenoids in fenugreek (*Trigonella foenum-graecum* L.): insights from integrated metabolomics and transcriptomics analysis

**DOI:** 10.3389/fpls.2025.1669610

**Published:** 2025-09-18

**Authors:** Shiqing Dong, Xiaokang Han, Xiuyuan Man, Xiangmin Deng, Zinuo Chen

**Affiliations:** ^1^ Jiangsu Key Laboratory for Eco-Agriculture Biotechnology Around Hongze Lake, School of Life Science, Huaiyin Normal University, Huai’an, Jiangsu, China; ^2^ School of Traditional Chinese Medicine, Jiangsu College of Nursing, Huai’an, Jiangsu, China

**Keywords:** fenugreek, metabolomics, transcriptomics, multi-omics, secondary metabolites, UPLC-MS/MS, ABC transporter, transcription factor

## Abstract

**Introduction:**

Fenugreek (*Trigonella foenum-graecum* L.) is a medicinal and edible plant containing bioactive compounds with therapeutic potential. However, the tissue-specific distribution and biosynthetic pathways of these compounds remain poorly characterized.

**Methods:**

This study employed metabolomics and transcriptomics to analyze metabolite profiles and gene expression in fenugreek roots, stems, and leaves. Using UPLC-MS/MS, we identified 2,124 metabolites, and full-length transcriptome sequencing alongside RNA-seq analysis revealed tissue-specific differentially expressed genes (DEGs).

**Results:**

Our analysis revealed distinct tissue-specific accumulation patterns of alkaloids, flavonoids, and terpenoids. The roots were identified as the primary site for the biosynthesis of these secondary metabolites. Multi-omics integration pinpointed key biosynthetic genes, such as *cytochrome P450s* (*CYP450s*) and *chalcone synthase* (*CHS*). Furthermore, integrated co-expression networks and molecular docking simulations indicated functional associations between ABC transporters and bioactive compounds. Transcriptional regulation analysis suggested MYB and bHLH transcription factors as potential modulators of metabolic biosynthesis.

**Discussion:**

This study enhances understanding of metabolic regulations in fenugreek and offers molecular resources for medicinal plant breeding.

## 1 Introduction

Fenugreek (*Trigonella foenum-graecum* L.), an annual self-pollinating plant of the Fabaceae family, is a resilient crop with cold tolerance and drought resistance. The plant is being widely cultivated across Asia, Africa, and the Mediterranean basin, including the northeastern, northwestern, and southwestern provinces of China (Naika et al., 2022; Zhao et al., 2024b). Originating from Iran and northern India, this multipurpose medicinal plant has gained importance not only as a culinary spice but also for its broad pharmacological properties. The plant is being used in the treatment of pulmonary disorders, lactation enhancement, glycemic regulation, and protection against heavy metal-induced hepatotoxicity (Odewumi et al., 2018; Almalki, 2022; Gonda et al., 2023; Sun et al., 2021). Additionally, emerging evidence underscores its multifaceted therapeutic applications, demonstrating efficacy in alleviating menopausal symptoms, inhibiting breast cancer cell proliferation, and mitigating neurodegenerative pathologies such as Alzheimer’s disease (Khanna et al., 2020; Khoja et al., 2022; Varshney and Siddique, 2023). Its functional food potential stems from a rich composition of dietary fiber, vitamins, and bioactive compounds, including flavonoids (rutin, vitexin), alkaloids (trigonelline), and saponins (diosgenin). These compounds collectively regulate metabolic functions, combat obesity, and exhibit antitumor, anti-inflammatory, and neuroprotective activities (Li et al., 2020; Avalos-Soriano et al., 2016). Notably, trigonelline and diosgenin show promise in addressing age-related muscle decline and cognitive dysfunction, while vitexin demonstrates anti-carcinogenic effects in colorectal and renal cancers (Aumsuwan et al., 2016; Wu et al., 2015; Membrez et al., 2024; Chen et al., 2024). These multifaceted bioactive properties position fenugreek as a valuable resource for advancing health interventions and longevity research.

The biosynthesis pathway of diosgenin in fenugreek has been substantially elucidated through recent research. Current evidence indicates that this metabolic route originates from the formation of isopentenyl diphosphate (IDP) precursors, which are subsequently converted to farnesyl pyrophosphate (FPP). This intermediate is then catalyzed by squalene synthase (SQS) to generate squalene. Subsequent enzymatic modifications involve squalene epoxidase (SEP) and cycloartenol synthase (CAS), which sequentially mediate the synthesis of cycloartenol through two-step epoxidation and cyclization processes (Nguyen et al., 2013; Gas-Pascual et al., 2014). The metabolic cascade proceeds with Δ24-reductase catalyzing the conversion to cholesterol, while C26-hydroxylase (C26-H) plays a pivotal role in the final biosynthetic steps yielding diosgenin (Mohammadi et al., 2020; Schaeffer et al., 2001; Nasiri et al., 2022). Notably, emerging studies have identified the cooperative involvement of cytochrome P450 monooxygenases and steroid-specific 3-*O*-glucosyltransferase in facilitating the cholesterol-to-diosgenin conversion, thereby refining our understanding of this metabolic network (Gao et al., 2021; Christ et al., 2019). However, it should be emphasized that the biosynthetic pathways governing other secondary metabolites in fenugreek, particularly flavonoids, terpenoids, and alkaloids, remain largely unexplored, with the associated enzymatic systems and regulatory mechanisms that remain to be systematically evaluated.

Elucidating the genetic regulatory networks that govern biochemical pathways is pivotal for establishing genotype-metabolotype correlations in plants that accumulate bioactive compounds (Salami et al., 2024, Salami et al., 2023; Riasat et al., 2018). Integrated metabolomic-transcriptomic approaches have emerged as powerful tools for deciphering the spatiotemporal dynamics of gene-metabolite interactions in plant secondary metabolism, particularly through multi-tissue comparative profiling. This study aims to (1) decipher tissue-specific regulatory networks governing alkaloid, flavonoid, and terpenoid biosynthesis in fenugreek, (2) identify key transporters and transcription factors (TFs) coordinating metabolite partitioning, and (3) establish genotype-metabolotype correlations to enable targeted bioengineering. To achieve these goals, we implemented an integrated multi-omics framework combining full-length transcriptomics and RNA-Seq profiling of root/stem/leaf tissues, UPLC-MS/MS-based metabolomic mapping, co-expression network analysis, and molecular docking validation. We anticipate this strategy will reveal novel biosynthetic hubs in roots, elucidate ABC transporter-mediated spatial distribution mechanisms, and provide genetic resources for enhancing medicinal compound production.

## 2 Materials and methods

### 2.1 Plant materials and growth condition

The fenugreek seeds used in this study were obtained from local germplasm resources in Lanzhou, Gansu Province, China. Species identification was performed through comprehensive analysis combining morphological characterization of plant specimens with DNA barcoding techniques targeting the *ITS2* and *psbA* genetic loci. Fenugreek seedlings were grown for one-month in a controlled chamber at 22°C with a 16/8-hour light/dark cycle. Root, stem, and leaf tissues were harvested for transcriptome and metabolome sequencing. Samples were immediately cryopreserved in liquid nitrogen to maintain biological activity. Three biological replicates were used for transcriptome analysis and five for metabolome analysis to ensure robustness.

### 2.2 Metabolomics profiling and data analysis

Metabolomics sample preparation and bioinformatics analysis were conducted at Shanghai Majorbio Co., LTD. (http://www.majorbio.com/) following standardized protocols. Briefly, 100 mg of plant samples were frozen, ground, and subjected to ultrasonic extraction at low temperature using 800 μL of extraction solvent (methanol:water (HPLC-grade, Sigma-Aldrich, USA) = 4:1, v:v) containing four internal standards (including L-2-chlorophenylalanine (CAS: 103616-89-3, Sigma-Aldrich, USA) at 0.02 mg/mL) to obtain the supernatant for analysis. Quality control (QC) samples were prepared by pooling equal volumes of sample extracts. One QC sample was inserted every 5-10 samples to monitor analytical repeatability. LC-MS/MS analysis was performed using the UHPLC-Q Exactive system (Thermo Fisher Scientific, USA). Chromatographic separation was performed using complementary ACQUITY UPLC BEH C18 (reversed-phase) and ACQUITY UPLC BEH Amide (hydrophilic interaction chromatography, HILIC) columns (100 mm × 2.1 mm i.d., 1.7 µm; Waters Corporation, USA) at 40°C with a flow rate of 0.40 mL/min. For reversed-phase separation, mobile phase A consisted of 2% acetonitrile (HPLC-grade, Fisher Chemical, USA) containing 0.1% formic acid (MS-grade, Sigma-Aldrich, USA) in water, and mobile phase B was acetonitrile with 0.1% formic acid. For HILIC separation, mobile phase A comprised 95% acetonitrile aqueous solution with 5 mM ammonium acetate (MS-grade, Sigma-Aldrich, USA), and mobile phase B was 5% acetonitrile in water containing 10 mM ammonium acetate. Mass spectrometry analysis was performed in both positive and negative ionization modes with a mass scan range of m/z 70-1050. Ion source parameters were set as follows: spray voltage at +3500 V (positive) and -3000 V (negative), sheath gas flow rate at 50 psi, auxiliary gas flow rate at 13 psi, and ion transfer tube temperature at 450°C. MS/MS fragmentation was carried out using stepped collision energies (20, 40, and 60 eV), with full-scan MS and MS/MS (MS) resolutions set to 70,000 and 17,500 (full width at half maximum, FWHM), respectively, to ensure accurate metabolite identification.

Raw data were processed using Progenesis QI (Waters Corporation, USA), and a data matrix was generated by matching against the self-constructed plant Metabolite Database (MJDBPM). Data pre-processing was conducted via cloud.majorbio.com, excluding variables in QC samples with a relative standard deviation (RSD) > 30%. The data were log10-transformed and analyzed using PCA and OPLS-DA via the R language ropls package (Version 1.6.2). Metabolites with VIP values > 1 and p-values < 0.05 (Student’s t-test) were considered significantly different. Metabolic pathways were annotated using the KEGG database, and pathway enrichment analysis was performed using the Python scipy.stats package. The most relevant biological pathways were identified through Fisher’s exact test.

### 2.3 Full-length library construction and SMRT sequencing

Total RNA was extracted from tissue samples using RNAprep Pure Plant Plus Kit (Polysaccharides & Polyphenolics-rich) (Tiangen Biotech, China) and quantified for concentration and purity using the Nanodrop 2000 spectrophotometer (Thermo Fisher Scientific, USA). RNA integrity was assessed via agarose gel electrophoresis, and the RNA integrity number (RIN) was determined using the Agilent 2100 Bioanalyzer. Equal amounts of RNA from roots, stems, and leaves were pooled. Full-length cDNA was synthesized using the SMARTer™ PCR cDNA Synthesis Kit (TaKaRa, Japan) and purified with PB magnetic beads (PacBio, USA) to construct the sequencing library. Library quality was rigorously evaluated before sequencing. Full-length transcriptome sequencing was performed on the PacBio platform (PacBio, USA). Post-sequencing, high-quality data were filtered through stringent quality control measures. Raw data were processed using Iso-Seq3 software (PacBio, USA) to generate full-length consensus sequences. Circular consensus sequencing (CCS) reads were extracted based on full passes ≥ 3 and sequence accuracy > 0.90. Data were further evaluated by analyzing circular consensus sequencing (CCS) sequence counts, base numbers, and average insert lengths.

### 2.4 Transcriptome sequencing analysis

The cDNA library was sequenced on the Illumina NovaSeq X Plus system (Illumina, USA). Raw sequencing data were processed using Fastp (Version 0.23.2) (https://github.com/OpenGene/fastp). Unigene sequences from RNA-Seq were aligned to SMRT-corrected transcript sequences as a reference. Annotated genes were compared against the nr, Swiss-Prot, Pfam, COG, GO, and KEGG databases. Gene expression levels were quantified using RSEM (Version 1.3.3) (http://deweylab.github.io/RSEM/). Differentially expressed genes (DEGs) were identified with DESeq2 (Version 1.38.3) (http://bioconductor.org/packages/stats/bioc/DESeq2/), applying thresholds of FDR < 0.05 and |log2FC| ≥ 1.

### 2.5 Integration analysis of metabolome and transcriptome

The biosynthesis mechanisms of fenugreek secondary metabolites were elucidated by integrating differentially expressed metabolites (DAMs) with differentially expressed genes (DEGs). We constructed a DEG-DAM association network based on KEGG functional pathways, enabling detailed statistical analysis of flavonoids, alkaloids, and terpenes, with a focus on their subclasses and metabolic pathways. Pearson correlation coefficients were calculated to quantify the relationship between metabolite levels and corresponding mRNA expression within each sample group. Correlation heatmaps and network maps were used to visualize these relationships.

### 2.6 Quantitative real-time PCR

To verify the accuracy of the transcriptome sequencing quantification results, we conducted qRT-PCR experiments. In the experimental methodology, we selected genes involved in metabolic pathways and validated their expression profiles via qRT-PCR. To ensure data precision and robustness, tissue samples were processed with a rigorous experimental design, including both biological and technical triplicates. Total RNA was extracted from the samples and reverse-transcribed into cDNA following standard protocols using the PrimeScript™ RT reagent Kit with gDNA Eraser (TaKaRa, Japan). qRT-PCR analysis was performed using the CFX96 real-time PCR detection system (Bio-Rad, USA) with SYBR Green master mix (AGBio, China). The *TfACT7* gene served as the internal reference for normalization. Gene expression levels were quantified using the 2^-ΔΔ^ *^C^* ^t^ method (Dong et al., 2024). Primer sequences and related details are provided in **Supplementary Table S1** to ensure reproducibility.

### 2.7 Molecule docking

To further verify the correlation between ABC transporters and metabolites, we conducted molecular docking experiments. The three-dimensional structure of the ABC transporter was predicted using AlphaFold2 (DeepMind, UK) and the model with the highest PLDDT score was selected. Ligand structures of plant secondary metabolites were retrieved from PubChem and optimized through energy minimization using Chem3D 19.0 (PerkinElmer, USA). Following binding pocket prediction with Proteins.plus (https://proteins.plus, ZBH Center for Bioinformatics, Germany), receptor protein preparation (water removal and hydrogenation) and ligand treatment (charge assignment) were performed using PyMol 4.6.0 (Schrödinger, USA) and AutoDockTools 1.5.6 (Scripps Research, USA), respectively. Blind docking was conducted with AutoDock Vina 1.1.2 (Scripps Research, USA), selecting the complex with the lowest binding free energy and hydrogen bond formation. Flexible docking refinement was subsequently performed focusing on amino acid residues within 5-Å of the ligand, guided by predicted active pocket localization. Final binding site characterization and interaction analysis were executed using PyMol (Schrödinger, USA) and LigPlot 2.2 (EMBL-EBI, UK), confirming the hydrogen bond-mediated ligand-amino acid residue binding mode (Peng et al., 2022; Ma et al., 2024).

### 2.8 Statistical analysis

Statistical analyses and graphical representations were performed using GraphPad Prism 6.0 (GraphPad Software, San Diego, CA). One-way analysis of variance (ANOVA) was conducted with SPSS v20.0 (IBM Corp., Armonk, NY). Gene expression levels and metabolite concentrations were visualized through heatmaps generated in Microsoft Excel 2019 using a tripartite color gradient in conditional formatting, with Z-score normalized data reflecting relative abundance patterns.

## 3 Results

### 3.1 Fenugreek metabolome profiling and DAMs identification

**Figure 1:**
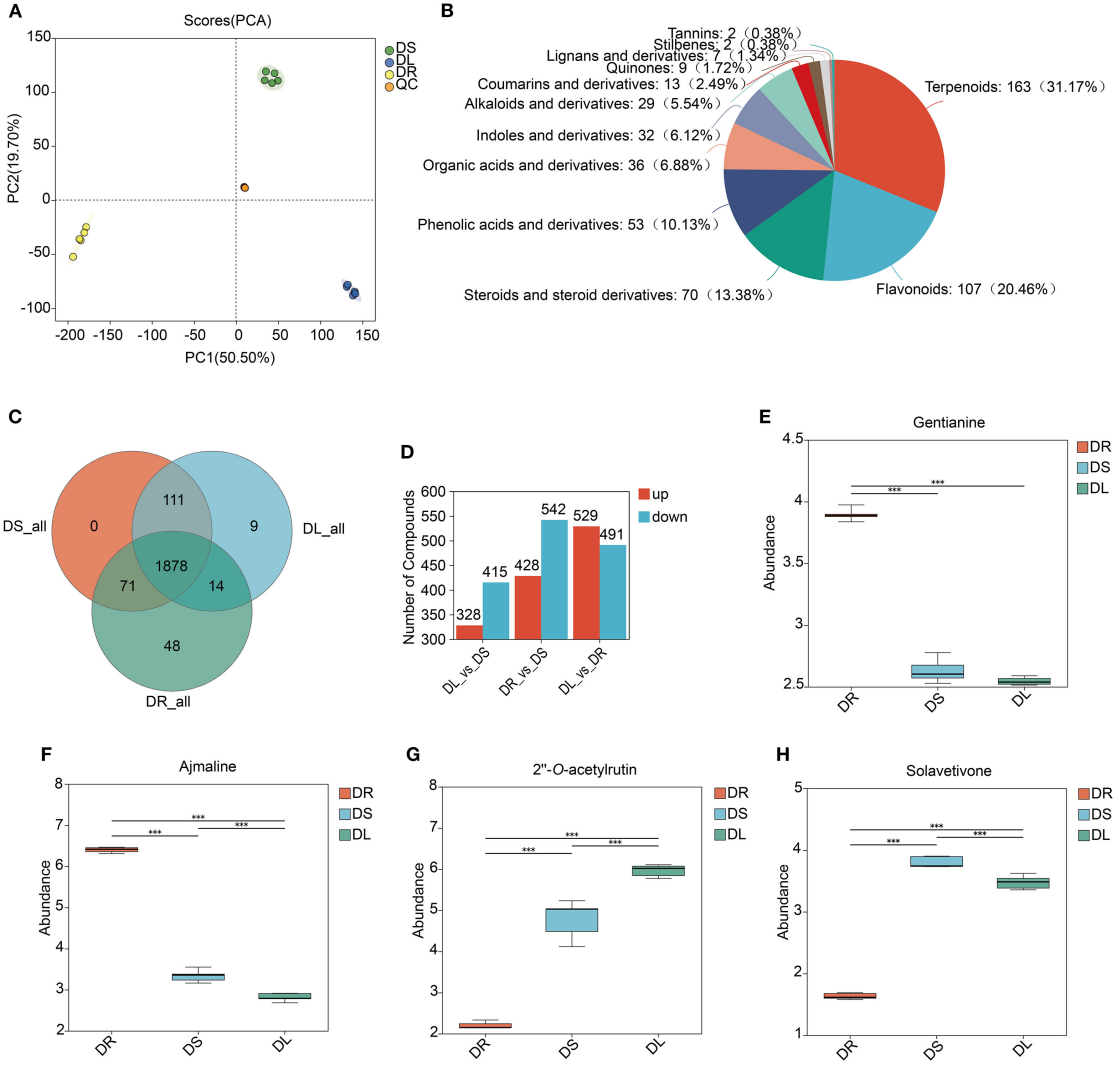
Metabolomic profiles of fenugreek. **(A)** PCA of the metabolomic data across multiple replicates. Diverse colors denote distinct tissues, with the inter-point distance indicating the relative tendency for separation or convergence. S: Stem; L: Leaf; R: Root. **(B)** The diversity of fenugreek secondary metabolites is depicted in pie charts, with the uniqueness and proportion of each type of metabolite arranged from highest to lowest by quantity. In the charts, different colors distinguish between various categories of metabolites, and the area of each color represents the relative proportion of metabolites within that category. **(C)** Venn Diagram illustrating the number of shared and unique metabolites across different tissues. **(D)** Statistical analysis of differential metabolites between different tissues, with red representing upregulation and blue representing downregulation. **(E-H)** Comparative content of gentianine, ajmaline, 2’’-*O*-acetylrutin and solavetivone among the three tissues. The vertical axis represents the relative abundance of each compound. The horizontal axis represents different tissues. *** indicates statistical significance at the level of p < 0.001.

To investigate the metabolomic profiles of fenugreek roots, stems, and leaves, we performed ultra-performance liquid chromatography-tandem mass spectrometry (UPLC-MS/MS) to establish a comprehensive metabolomic database. Through PCA, we observed a significant reduction in variability between biological replicates, which validated the consistency and reliability of the experimental results. PCA results also revealed distinct metabolomic differences among the tissues (**Figure 1A**). After data preprocessing, 1476 and 648 metabolites were identified in positive and negative ion modes, respectively. Phytochemical classification annotated 327 primary metabolites (29% of total), 523 secondary metabolites (46%), and 281 unclassified metabolites (25%) (**Supplementary Figure S1**). Among secondary metabolites, terpenoids (163 types, 31.17%), flavonoids (107 types, 20.46%), and steroids/derivatives (70 types, 13.38%) were predominant (**Figure 1B**). Metabolite analysis detected 2011, 2060, and 2012 metabolites in roots, stems, and leaves, respectively, with 1878 shared among all three tissues (**Figure 1C**), indicating high metabolic similarity across tissues.

To identify tissue-specific metabolic regulation, we performed pairwise comparisons and identified differentially accumulated metabolites (DAMs). The comparison between leaves and roots (DL_vs_DR) showed the most pronounced differences, with 1,020 DAMs, followed by root vs. stem (DR_vs_DS, 970 DAMs) and leaf vs. stem (DL_vs_DS, 743 DAMs) (**Figure 1D**). Notably, the number of up- and down-regulated metabolites in each comparison was 529/491, 428/542, and 328/415, respectively. KEGG pathway enrichment analysis of tissue-specific DAMs uncovered key metabolic specializations in each organ. In roots, the 374 unique DAMs were significantly enriched in pathways for amino acid metabolism, as well as the biosynthesis of isoflavonoids and alkaloids (**Supplementary Figures S2A, B**). Conversely, stems harbored 110 specific DAMs, which were primarily linked to betaine biosynthesis and the metabolism of phenylalanine and tryptophan (**Supplementary Figures S2C, D**). Finally, leaves exhibited 245 unique DAMs, with enrichment strongly pointing to pathways in flavonoid/flavonol biosynthesis and arginine/proline metabolism (**Supplementary Figures S2E, F**). We also identified shared DAMs between tissues included: Roots and stems: 220 DAMs, enriched in isoflavonoid and flavonoid biosynthesis, tryptophan metabolism, and alpha-linoleic acid metabolism (**Supplementary Figures S2G, H**). Stems and leaves: 398 DAMs, associated with flavonoid and flavonol biosynthesis, glutathione metabolism, tryptophan and phenylalanine metabolism, TCA cycle, betaine biosynthesis, branched-chain amino acid biosynthesis, isoflavonoid biosynthesis, and linoleic acid metabolism (**Supplementary Figures S2I, J**). Roots and leaves: 23 DAMs, primarily involved in one-carbon pool by folate, N-glycan biosynthesis, lipoic acid metabolism, cyanoamino acid metabolism, and glycine, serine, and threonine metabolism (**Supplementary Figures S2K, L**). These findings demonstrate distinct metabolite distribution patterns across tissues. For example, alkaloids (e.g., gentianine and ajmaline) were predominantly stored in roots, while flavonoids (e.g., 2’’-*O*-acetylrutin) and terpenoids (e.g., solavetivone) were significantly enriched in stems and leaves (**Figures 1E-H**).

### 3.2 Characterization of the full-length transcriptome of fenugreek

In the absence of a reference genome, we employed single-molecule real-time sequencing (SMRT) technology to sequence the full-length transcriptome of fenugreek, generating approximately 37.8 Gb of sequencing data. After self-correction and data curation, we obtained 228,601 circular consensus sequences (CCSs), of which 203,123 were identified as full-length non-chimeric (FLNC) reads for subsequent analysis (**Supplementary Table S2**). The length of FLNC reads ranged from 80 to 10,636 base pairs (bp), with an average length of 1,696 bp and an N50 length of 1,900 bp (**Supplementary Figure S2**). We then performed redundancy removal and completeness assessment on these transcripts. Furthermore, we annotated the FLNC reads with functional annotations from the NR, Uniprot, GO, KEGG, and Pfam databases, annotating 45,863 (93.71%), 45,907 (93.80%), 20,035 (40.94%), 14,317 (29.30%), and 16,210 (33.12%) genes, respectively (**Supplementary Table S3**).

### 3.3 Transcriptome analysis of DEGs in fenugreek

**Figure 2:**
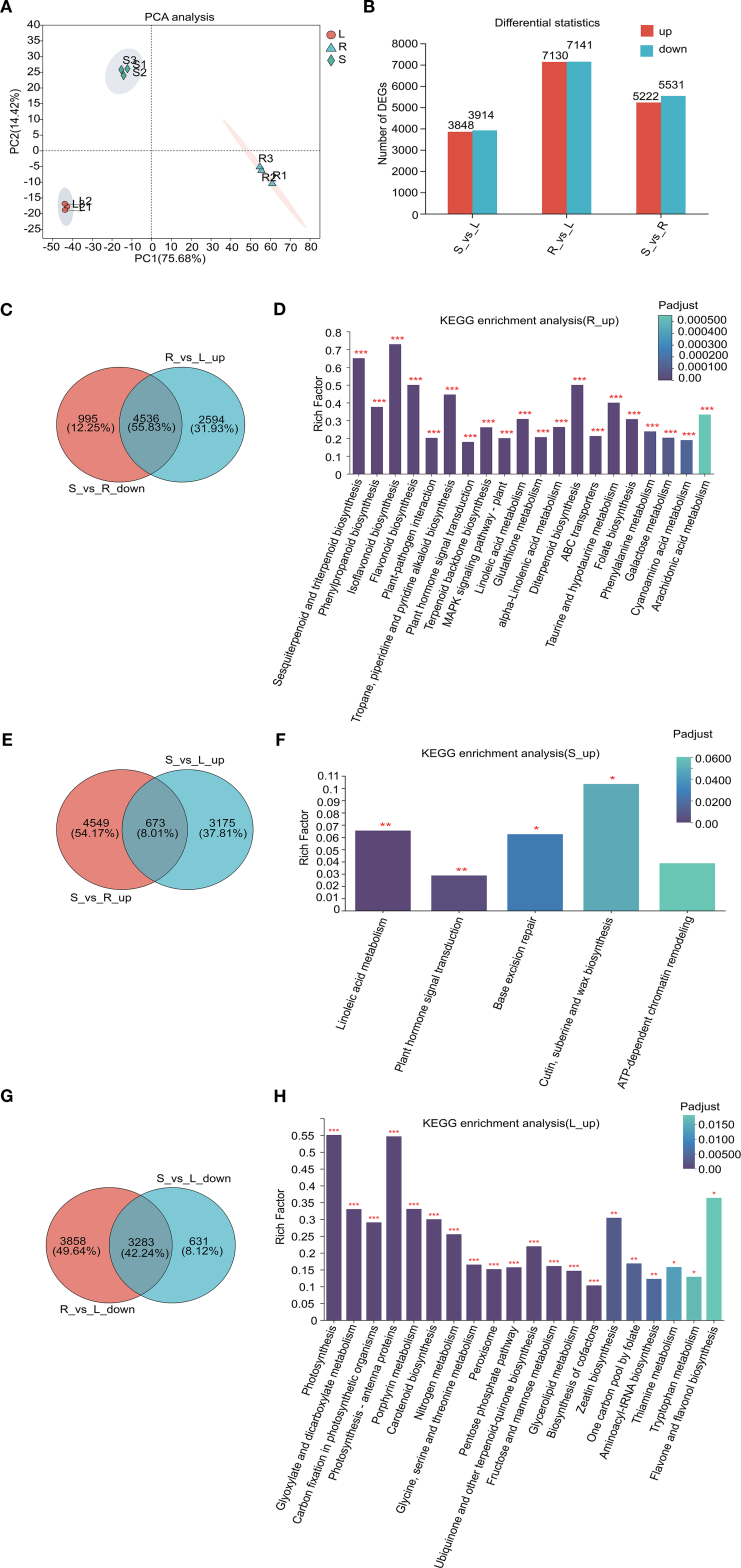
Comprehensive analysis of transcriptome and DEGs in different tissues. **(A)** PCA plots depicting the relationships between samples; **(B)** A statistical map of expression differences, where the horizontal axis represents distinct groups of differential expression comparisons, and the vertical axis indicates the corresponding counts of up-regulated and down-regulated genes. Red denotes up-regulated genes, while green signifies down-regulated genes. **(C, E, G)** Venn diagrams illustrating tissue-specific gene expression. The circles in different colors correspond to separate gene sets, with numerical values within the circles representing the counts of shared and unique genes across these sets. **(D, F, H)** KEGG pathway analysis of tissue-specific expression genes, with the horizontal axis representing the KEGG pathways and the vertical axis representing the Rich factor. An increased Rich factor indicates a higher level of enrichment. The histogram’s color gradient reflects the significance of enrichment, with Padjust < 0.001 indicated by ***, Padjust < 0.01 by **, and Padjust < 0.05 by *.

To investigate DEGs among fenugreek tissues (roots, stems, and leaves), we extracted transcriptome data, generating a comprehensive dataset of 58.54 Gb high-quality clean data. Each sample exceeded 5.9 Gb with a Q30 base percentage >95.98% (**Supplementary Table S4**). Using full-length transcriptome data as a reference, we performed *de novo* assembly, identifying 48,939 unigenes with an average N50 length of 2,172 bp (**Supplementary Figure S4A**). Clean reads were aligned to the assembled sequences, yielding mapping rates of 76.82%-80.33% (**Supplementary Figure S4B**). Correlation analysis confirmed biological replicate reliability, with Pearson correlation coefficients (r) ranging from 0.148 to 1 (**Supplementary Figure S4C**). PCA highlighted significant transcriptomic variations between tissues (**Figure 2A**).

To identify tissue-specific gene expression patterns, we screened for DEGs using thresholds of Padjust < 0.05 and |log2Ratio| ≥ 2. Pairwise comparisons revealed 10,753 DEGs in stem vs. root (S_vs_R; 5,222 upregulated, 5,531 downregulated), 14,271 DEGs in root vs. leaf (R_vs_L; 7,130 upregulated, 7,141 downregulated), and 7,762 DEGs in stem vs. leaf (S_vs_L; 3,848 upregulated, 3,914 downregulated) (**Figure 2B**). Venn diagram and KEGG pathway analyses highlighted tissue-specific gene expression patterns and their associated metabolic pathways. In roots, 4,536 high-expression genes were enriched in pathways such as flavonoid biosynthesis, terpenoid backbone biosynthesis, tropane, piperidine and pyridine alkaloid biosynthesis, plant hormone signal transduction, and ABC transporters (**Figures 2C, D**). In contrast, stems exhibited 673 high-expression genes linked to linoleic acid metabolism, plant hormone signal transduction, and cutin, suberine and wax biosynthesis (**Figures 2E, F**). Leaves showed 3,283 high-expression genes primarily involved in photosynthesis, nitrogen metabolism, glycine, serine and threonine metabolism, and fructose and mannose metabolism pathways (**Figures 2G, H**). Additionally, shared high-expression genes were identified: 2,587 in roots and stems (enriched in phenylpropanoid biosynthesis, DNA replication, and chromatin remodeling) (**Supplementary Figures S5A, B**), 369 in roots and leaves (associated with ABC transporters, arginine and proline metabolism, and monoterpenoid biosynthesis) (**Supplementary Figures S5C, D**), and 4,342 in stems and leaves (involved in photosynthesis, amino acid metabolism, and peroxisome pathways) (**Supplementary Figures S5E, F**). Collectively, these results indicate that roots serve as the primary site for the synthesis of various secondary metabolites in fenugreek, and suggest that ABC transporters may play a crucial role in the tissue-specific distribution of flavonoids, terpenoids, and alkaloids.

### 3.4 Integrated transcriptomic and metabolomic analysis

To decipher the biosynthetic pathways of key medicinal compounds in fenugreek, we integrated transcriptomic and metabolomic data. Orthogonal two-partial least squares (O2PLS) regression revealed high loading values for both genes and metabolites, indicating strong congruence between the datasets (**Supplementary Figure S6A**). Based on annotation information, we comprehensively analyzed genes and metabolites associated with three major classes of secondary metabolites: flavonoids, alkaloids, and terpenoids.

**Figure 3:**
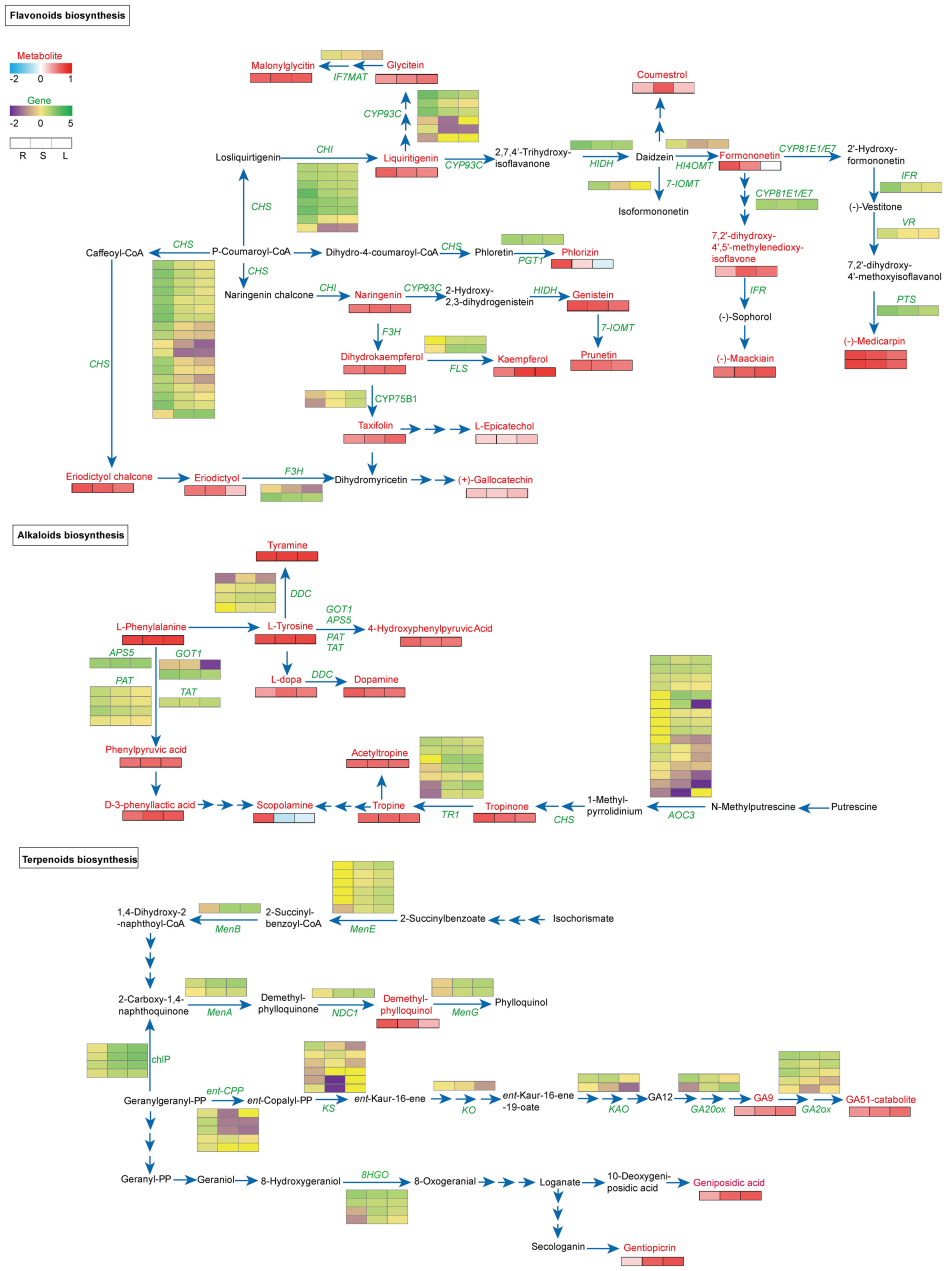
Analysis of the biosynthesis and expression network of flavonoids, alkaloids, and terpenoids. Significantly enriched genes identified by the hypergeometric distribution algorithm are highlighted in green, while metabolites are highlighted in red. In the figure, red or blue squares indicate an increase or decrease in metabolites, respectively, and green or purple squares indicate an increase or decrease in gene expression, respectively.

Flavonoid biosynthesis analysis identified 5 key KEGG pathways (**Supplementary Figures S6B, C**). We then constructed a co-expression network linking gene expression with metabolite accumulation across tissues (**Figure 3**). Within this network, upregulated genes and highly abundant metabolites are denoted by green and red squares, respectively, while downregulated genes and low-abundance metabolites are marked by purple and blue squares. Specifically, 18 DEGs encoding CHS were identified, with only *All_1_transcript_6824* was found to be up-regulated in stems and leaves, while the others were up-regulated in roots. This likely explains the significant accumulation of eriodictyol chalcone and eriodictyol in roots. Similarly, 4 CHI-encoding genes (*All_1_transcript_78079*, *_72877*, *_81643*, and *_86801*) were significantly upregulated in roots, potentially leading to substantial liquiritigenin enrichment. In leaves, two TfCYP75B1 genes (*All_1_transcript_64340* and *_60598*) were upregulated, possibly contributing to elevated taxifolin content. Concurrently, two *flavonol synthase* (*FLS*) were upregulated in stems and leaves, explaining the high kaempferol levels in these tissues. Additionally, several genes (*7-IOMT*, *PTS*, *VR*, *IFR*, and *HI4OMT*) were predominantly upregulated in roots, correlating with higher enrichment of prunetin, formononetin, and (-)-medicarpin in this tissue (**Figure 3**; **Supplementary Tables S5**, **S6**).

For alkaloid biosynthesis, we identified seven *TfTR1* (*tropinone reductase*) with differential expression across tissues. Four transcripts (*All_1_transcript_82660*, *_21066*, *_83660*, and *_85835*) were significantly upregulated in stems and leaves, while one (*All_1_transcript_81371*) was upregulated in roots—consistent with scopolamine accumulation primarily in roots. Four *DDCs* (*tyrosine/DOPA decarboxylase*) showed tissue-specific expression patterns inversely correlating with dopamine enrichment in roots. All four *TfPATs* (*glutamate/aspartate-prephenate aminotransferase*) were upregulated in roots, while 16 *AOCs* (*amine oxidase copper containing*) showed differential expression across tissues (**Figure 3**; **Supplementary Tables S5**, **S6**).

In terpenoid analysis, diterpenoids such as GAs were highly enriched in leaves, inversely correlating with *GA20ox* (*All_1_transcript_70676*, encoding gibberellin 20-oxidase) and *GA2ox* (*All_1_transcript_14062*, *_42566*, *_76748*) expression. Conversely, demethylphylloquinol was significantly enriched in roots, negatively associated with *TfMenA* (*All_1_transcript_8332*, *_50566*, encoding 2-carboxy-1,4-naphthoquinone phytyltransferase) and *NDC1* (*All_1_transcript_57676*, encoding pyridine nucleotide-disulphide oxidoreductase) expression. Monoterpenoids including gentiopicroside and geniposidic acid were enriched in stems and leaves, consistent with elevated *MTD* (*All_1_transcript_72649*, *_74582*, encoding mannitol dehydrogenase) expression, suggesting its regulatory role in monoterpenoid biosynthesis (**Figure 3**; **Supplementary Tables S5**, **S6**). Finally, we validated the tissue-specific expression patterns of key genes (*TfCYP75B1*, *TfCHI*, *TfCHS*, *TfTR1*, *TfPAT*, and *TfMenA*) by qRT-PCR, which strongly correlated with transcriptome sequencing data (**Supplementary Figure S7**), confirming the reliability of our findings.

### 3.5 Correlation and molecular docking between DAMs and ABC transporters

**Figure 4:**
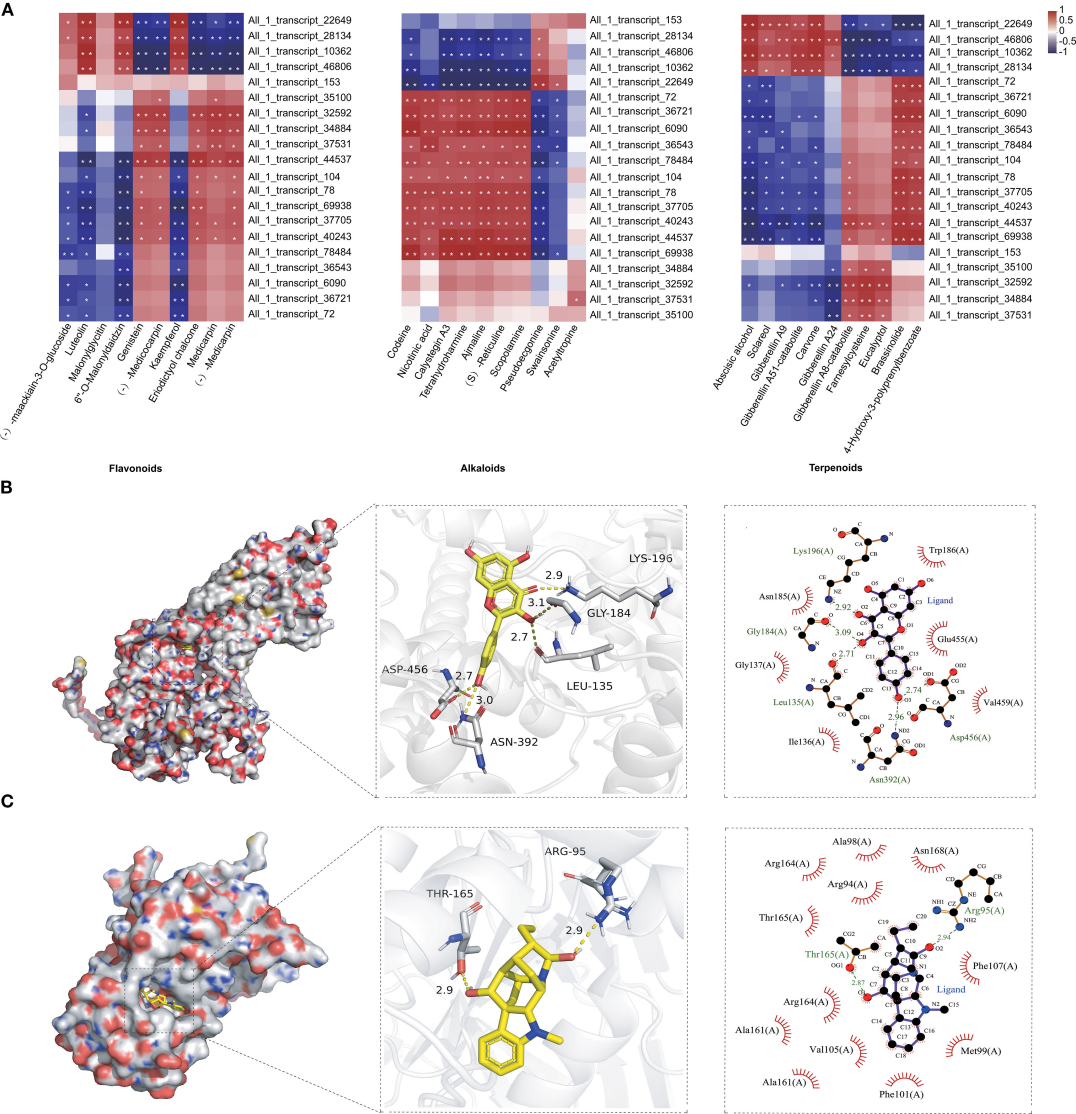
Correlation analysis and molecular docking between ABC transporters and secondary metabolites of fenugreek. **(A)** Each row corresponds to a gene, and each column corresponds to a metabolite. The individual cells within the diagram indicate the correlation strength between the respective genes and metabolites. Correlations with P < 0.01 are denoted by **, and those with P < 0.05 are marked by *. **(B)** Molecular docking analysis of TfABCG11 (*All_1_transcript_22649*) with kaempferol. **(C)** Molecular docking analysis of TfABCI17 (*All_1_transcript_69938*) with ajmaline. From left to right, the panels depict the ligand-protein binding within the pocket, the specific interactions with amino acid residues, and the detailed force analysis of the ligand-protein interaction.

ABC transporters, one of the largest and most ancient protein families, play a pivotal role in the transport of secondary metabolites such as flavonoids, terpenoids, and alkaloids in plants (Zhao et al., 2024a; Ichino and Yazaki, 2022; Li et al., 2024; Lin et al., 2024). This study highlights their potential role in the tissue-specific distribution of fenugreek metabolites (**Figure 2**; **Supplementary Figure S5**). We identified 318 ABC transporter genes in fenugreek, of which 178 exhibited tissue-specific differential expression (**Supplementary Figure S8A**). These genes were grouped into 10 clusters based on expression profiles. Clusters 1 and 4 contained 64 and 28 DEGs, respectively, upregulated in roots or stems. Cluster 2 included 22 DEGs that were upregulated in both roots and leaves. Cluster 5 comprised 7 DEGs that were exclusively upregulated in stems. Clusters 3 and 6 contained 39 and 14 DEGs, respectively, upregulated in stems or leaves (**Supplementary Figure S8B**). Correlation analysis between the top 20 ABC transporter genes and metabolites (flavonoids, alkaloids, and terpenoids) revealed significant positive or negative associations (**Figure 4A**). To further elucidate the interactions between metabolites and ABC transporters, molecular docking studies were conducted (**Supplementary Table S7**). Specifically, TfABCG11 (*All_1_transcript_22649*) exhibited strong binding affinity with kaempferol, forming 5 hydrogen bonds (Leu135, Gly184, Lys196, Asn392, Asp456) and a binding free energy of -8.1 kcal/mol (**Figure 4B**). Similarly, TfABCI17 (*All_1_transcript_69938*) formed 2 hydrogen bonds with ajmaline (Arg95, Thr165), with a binding free energy of -11.0 kcal/mol (**Figure 4C**). These results demonstrate favorable binding affinities, providing a foundation for further functional studies.

### 3.6 Correlation between DAMs and TFs

**Figure 5:**
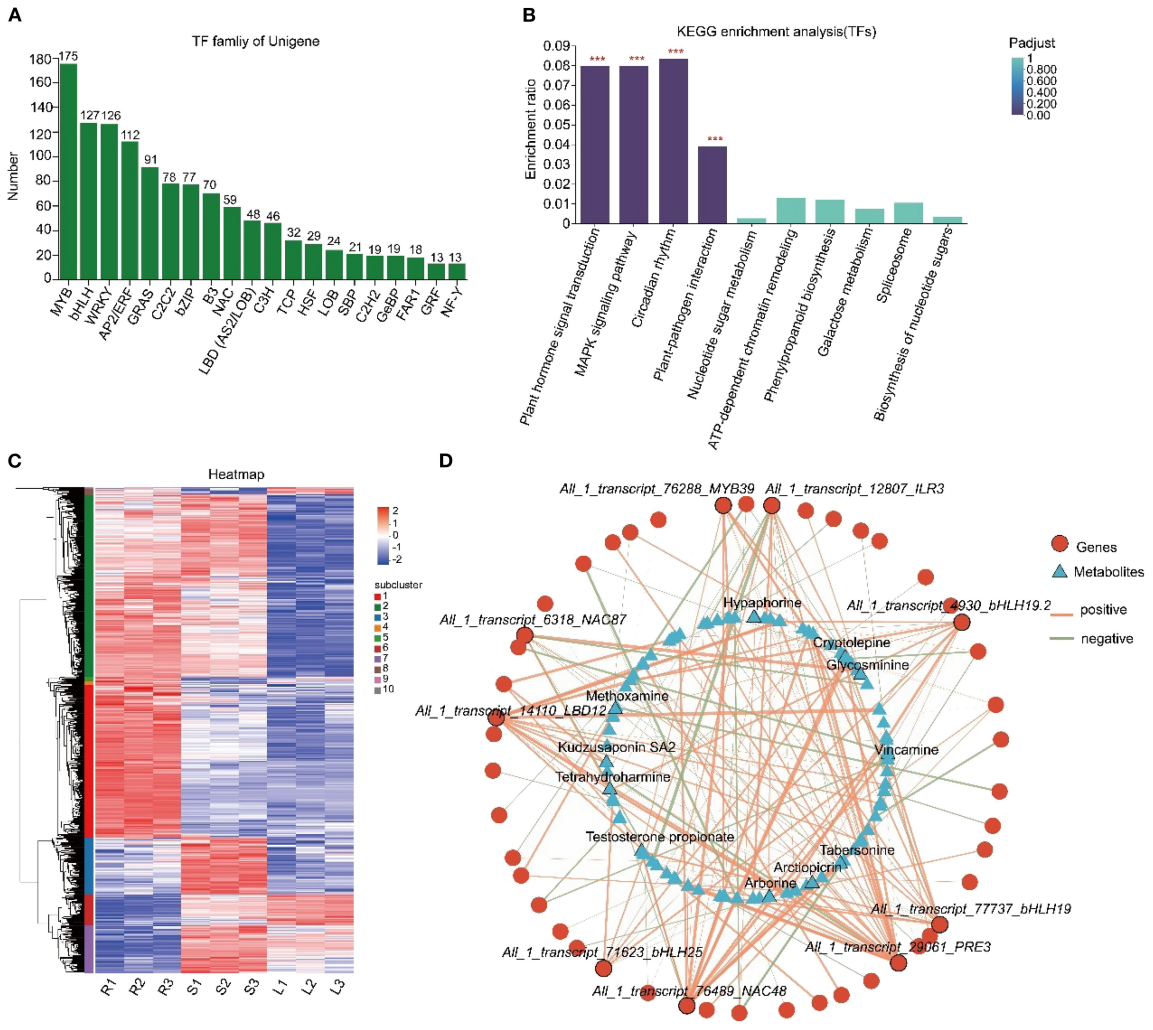
Identification of *TFs* and co-expression network analysis with DAMs. **(A)** Statistics of TF families. The x-axis represents different TF families, while the y-axis indicates the number of unigenes belonging to each family. **(B)** KEGG enrichment analysis of TFs. The x-axis denotes KEGG pathways, and the y-axis represents the Rich factor. A higher value signifies a more significant enrichment level. Histogram color gradients reflect the significance of enrichment, with “***” indicating Padjust < 0.001. **(C)** Clustering heatmap analysis of *TFs*. Each column corresponds to a sample, and each row represents a unigene. The color intensity indicates the expression level of the unigene in the sample, with red representing high expression and blue representing low expression. The specific change trend of the value can be referred to by the digital label of the color bar in the upper right corner. The dendrogram on the left is the unigene cluster diagram, where closer branch spacing indicates more similar expression levels. **(D)** Co-expression network analysis of TFs and DAMs. Triangles represent metabolites, circles represent genes, and lines represent correlations. Red lines indicate positive correlations (cor > 0), while green lines indicate negative correlations (cor < 0). The thickness of the lines is proportional to the absolute value of the correlation coefficient, meaning that thicker lines correspond to greater absolute values of the correlation coefficient, and thinner lines correspond to smaller absolute values.

Transcriptional regulation constitutes a pivotal component of gene expression control. Understanding the role of TFs in the biosynthesis of active ingredients in medicinal plants is crucial for developing novel plant varieties (Zheng et al., 2023). Our analysis of transcriptome sequencing data revealed 1287 TFs, with significant enrichment observed in the MYB, bHLH, WRKY, AP2/ERF, GRAS, and C2C2 families (**Figure 5A**). KEGG analysis revealed their enrichment in pathways related to plant hormone signal transduction, MAPK signaling pathway, circadian rhythm, and plant-pathogen interactions (**Figure 5B**). Furthermore, 817 TFs showed tissue-specific expression patterns (**Figure 5C**) and were grouped into 10 clusters. Cluster 1 comprised 258 TFs upregulated in roots. Cluster 2 included 306 TFs upregulated in both roots and stems. Cluster 3 encompassed 96 TFs exclusively upregulated in stems. Cluster 6 contained 52 TFs upregulated in leaves. Cluster 7 comprised 79 TFs upregulated in both stems and leaves (**Supplementary Figure S9**). Co-expression network analysis identified key gene-metabolite correlations. For instance, the biosynthesis of hypaphorine, tabersonine and glycosminine appears to be regulated by *All_1_transcript_76288_MYB39*, *All_1_transcript_6318_NAC87* and *All_1_transcript_76489_NAC48*. The biosynthesis of vincamine may be regulated by *All_1_transcript_76288_MYB39* and *All_1_transcript_71623_bHLH25* (**Figure 5D**).

## 4 Discussion

### 4.1 Tissue-specific metabolic compartmentalization in fenugreek

Fenugreek, a versatile culinary and medicinal plant, derives its therapeutic properties primarily from its diverse secondary metabolites. This study employed UPLC-MS/MS to construct a comprehensive metabolomics database of fenugreek, uncovering significant metabolic variations across its root, stem, and leaf tissues. Our analysis identified numerous DAMs within these tissues. Notably, the roots of fenugreek were found to be the primary reservoirs for flavonoids, alkaloids, and terpenoids, whereas the leaves accumulated specific flavonoids and diterpenoids (**Figure 1**). This suggests tissue-specific specialization in metabolite distribution, likely related to the plant’s medicinal properties. This pattern resembles strategies in other medicinal legumes. For example, in *Pueraria lobata*, both roots and stems synthesize puerarin (Xi et al., 2023). However, while *Pueraria lobata* produces its key isoflavone in multiple organs, fenugreek accumulates most bioactive compounds mainly in the roots, highlighting an evolutionary divergence in metabolic regulation within legumes. Furthermore, DEG analysis supported this hypothesis, showing elevated expression of genes involved in secondary metabolite biosynthesis, particularly in roots (**Figure 2**). These results align with previous studies (Naika et al., 2022).

### 4.2 *De novo* transcriptome profiling reveals biosynthetic drivers

In the absence of a reference genome for fenugreek, full-length transcriptome sequencing provided a comprehensive overview of expressed genes (**Supplementary Figure S2**). While previous studies have documented transcriptomic variations across different fenugreek tissues (Naika et al., 2022), a metabolomics-integrated analysis to identify key regulatory genes in the biosynthesis of critical metabolites has been lacking. Our transcriptomic profiling revealed numerous DEGs linked to secondary metabolite biosynthesis. Notably, the upregulation of genes encoding pivotal enzymes in the biosynthesis of flavonoids, alkaloids, and terpenoids suggests a potential correlation between gene expression and metabolite accumulation (**Figure 3**). Diosgenin, a significant secondary metabolite in fenugreek, has well-documented biosynthetic pathways involving TfS3GT2 (sterol-specific glucosyltransferase) and TfCYP90B50, which catalyzes cholesterol oxidation to produce diosgenin (Gao et al., 2021; Christ et al., 2019). However, pathways for other valuable metabolites remain unexplored. For instance, eriodictyol, a flavonoid with diverse biological activities (Islam et al., 2020), is synthesized via key enzymes such as CHS, CHI, and F3’H/CYP (Wu et al., 2022). Our study detected significant enrichment of eriodictyol in fenugreek roots and identified potential involvement of *All_1_transcript_6824_TfCHS*, *TfCHI* (*All_1_transcript_78079*, *All_1_transcript_72877*, *All_1_transcript_81643*, *All_1_transcript_86801*), and *TfCYP75B1* (*All_1_transcript_64340*, *All_1_transcript_60598*) in its biosynthesis (**Figure 3**). Similarly, scopolamine, a tropane alkaloid with sedative and anti-cholinergic properties (Chen et al., 2022), is synthesized via TR, a key rate-limiting enzyme (Rasi et al., 2024). Our findings reveal significant enrichment of scopolamine in fenugreek roots, with one *TR* (*All_1_transcript_81371*) being markedly up-regulated in this tissue (**Figure 3**). Gentiopicroside, known for its anti-inflammatory and antioxidant properties, has been reported in *Gentiana dahurica* (Li et al., 2022; Cao et al., 2016), but its biosynthesis in fenugreek remains uncharacterized. Our study identified gentiopicroside enrichment in fenugreek leaves and stems, with *MTD* (*All_1_transcript_72649*, *All_1_transcript_74582*) expression positively correlated, suggesting its potential role in biosynthesis (**Figure 3**).

### 4.3 Evolutionarily conserved roles of ABC transporters in metabolite partitioning

Plant secondary metabolites, particularly flavonoids, terpenoids, and alkaloids, are synthesized and accumulate in specific tissues. Transport mechanisms are essential in this process. For instance, AaPDR3 in *Artemisia annua* transports sesquiterpene β-caryophyllene within trichomes (Fu et al., 2017). SmABCG1, localized in the plasma membrane, facilitates tanshinone transport from root peridermic cells in *Salvia miltiorrhiza* (Li et al., 2024). CjMDR1 transports berberine from root tissue to root cells for storage in *Coptis japonica* (Shitan et al., 2003). CaABCG14 is associated with changes in capsaicin concentration in pepper septum and co-expresses with capsaicin biosynthesis genes. Gene silencing and overexpression studies show that CaABCG14 regulates capsaicin accumulation (Fei et al., 2024). This study revealed that ABC transporters play a crucial role in the tissue-specific distribution of fenugreek metabolites (**Figure 2**, **Supplementary Figure S5**). We identified 178 tissue-specific ABC transporter genes and conducted cluster analysis based on their expression levels (**Supplementary Figure S6**). Co-expression heat maps illustrated significant correlations between ABC transporter genes and metabolites (**Figure 4**). Furthermore, molecular docking predicted direct interactions between ABC transporters and metabolites at the protein structural level (**Figures 4B, C**). These transporters may modulate metabolite transport and distribution across tissues.

### 4.4 Uncharted transcriptional landscape governing bioactive compound synthesis

In recent years, the transcriptional regulation of secondary metabolite biosynthesis in medicinal plants has garnered extensive attention. Notable advancements have been achieved in elucidating the regulatory mechanisms of key compounds, such as artemisinin, taxol, tanshinone, ginsenosides, camptothecin, and salvianolic acid (Shi et al., 2024). The role of MYB, bHLH, NAC and WRKY families in the regulation of secondary metabolites are well-documented. For instance, in artemisinin biosynthesis, TFs such as AaMIXTA1, AaMYC2, AabZIP1, and AabHLH113 exert crucial regulatory influences (Shi et al., 2018; Shen et al., 2016; Yuan et al., 2023; Zhang et al., 2015). Similarly, TFs such as SmbHLH53, SmMYB12, SbMYB45 and SbMYB86.1 have been shown to play a crucial role in the synthesis and regulation of flavonoids (Fang et al., 2023; Wang et al., 2022; Peng et al., 2020), while OpNAC1, OpMYB1, and OpWRKY3 are essential for camptothecin biosynthesis (Hao et al., 2023; Rohani et al., 2016; Wang et al., 2019). This regulatory paradigm is also evident in legumes. In soybean (*Glycine max*), multiple MYB TFs (e.g., GmMYB29/39/100/133/176) have been demonstrated to positively or negatively regulate isoflavonoid accumulation (Yan et al., 2015; Liu et al., 2013; Chu et al., 2017; Bian et al., 2018). Likewise, in *Medicago sativa*, MsMYB206 directly transcriptionally activates key flavonoid biosynthetic genes, *MsFLS* and *MsF3’H* (Su et al., 2025). Furthermore, in *Pueraria lobata* var. *thomsonii*, the expression of several *PtMYBs* is closely correlated with puerarin biosynthesis (Wu et al., 2023), underscoring a conserved role for MYB TFs in regulating specialized metabolism across leguminous species. Despite these advances, the transcriptional regulation of key secondary metabolites in fenugreek remains largely unexplored. Through systematic identification, 1287 TFs were identified, with 817 showing tissue-specific expression (**Figure 5A, C**). Further co-expression network analysis revealed the close correlation between potential TFs and metabolites, providing a new perspective and valuable insights into the biosynthetic regulatory mechanisms of bioactive compounds in fenugreek (**Figure 5D**).

## 5 Conclusion

Fenugreek’s secondary metabolites hold significant potential for pharmaceutical and nutritional applications. In this study, we used an integrated metabolomics and transcriptomics approach to analyze DAMs and DEGs in fenugreek seedling tissues (roots, stems, and leaves). Our findings revealed that the roots are the primary site for synthesizing and accumulating most secondary metabolites, while specific terpenoids and flavonoids were mainly enriched in stems and leaves. Through multi-omics analysis, we identified key enzyme-coding genes involved in the biosynthesis of alkaloids, flavonoids, and terpenoids, providing essential genetic resources for engineering high-efficiency medicinal compound production. Additionally, we discovered ABC transporter genes and TFs potentially regulating secondary metabolite biosynthesis and transport. Co-expression networks and molecular docking elucidate metabolite-transporter interactions. These findings provide genetic resources for engineering high-yield metabolite production and support molecular breeding strategies for pharmaceutical applications.

## Data Availability

The datasets presented in this study can be found in online repositories. The names of the repository/repositories and accession number(s) can be found in the article/**Supplementary Material**.
