# Supplementary material for: Tissue-specific biosynthesis and regulation of alkaloids, flavonoids, and terpenoids in fenugreek (Trigonella foenum-graecum L.): insights from integrated metabolomics and transcriptomics analysis
Source: Front Plant Sci. 2025 Sep 18;16:1669610. doi: 10.3389/fpls.2025.1669610 (PMC12488739; doi:10.3389/fpls.2025.1669610)
Supplement: Supplementary file 1 [file DataSheet1.docx]

**Supplementary Information**


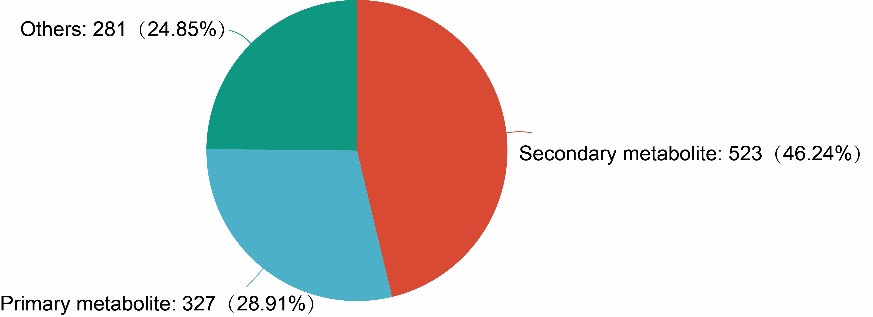


Fig. S1. Pie chart depicting the classification of plant metabolites.

The distinct colors in the pie chart correspond to the categorization of metabolites from various plant compounds, with the area of each color segment reflecting the relative abundance of metabolites within that particular classification.


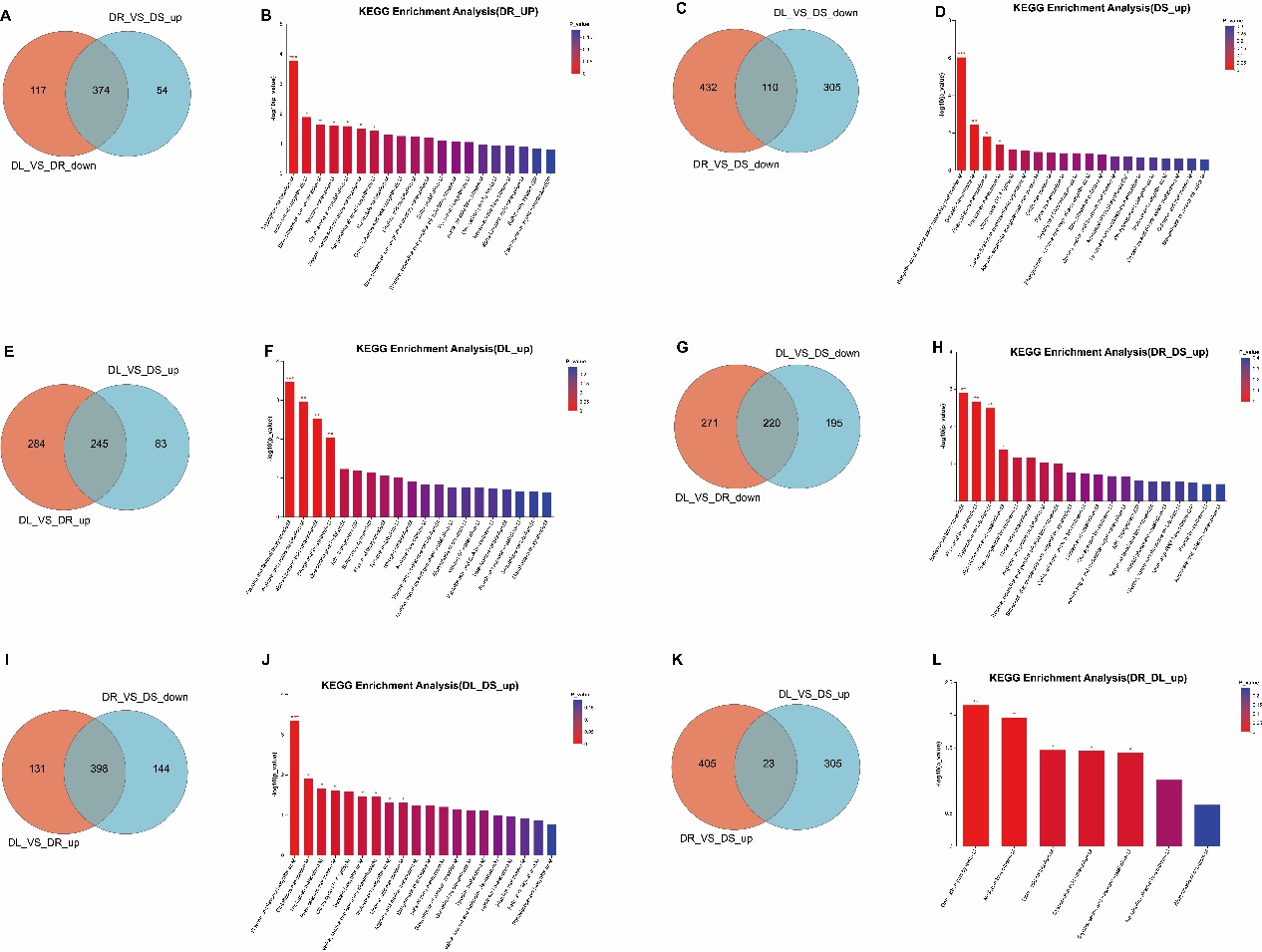


Fig. S2. Venn and KEGG analyzed the tissue distribution of fenugreek metabolites

(A, C, E, G, I, K) Venn diagrams illustrating tissue-specific metabolites. The circles in different colors correspond to separate metabolite sets, with numerical values within the circles representing the counts of shared and unique metabolites across these sets.

(B, D, F, H, J, L) KEGG pathway analysis of tissue-specific metabolites, with the horizontal axis representing the KEGG pathways and the vertical axis representing the Rich factor. An increased Rich factor indicates a higher level of enrichment. The histogram's color gradient reflects the significance of enrichment, with Padjust < 0.001 indicated by ***, Padjust < 0.01 by **, and Padjust < 0.05 by *.


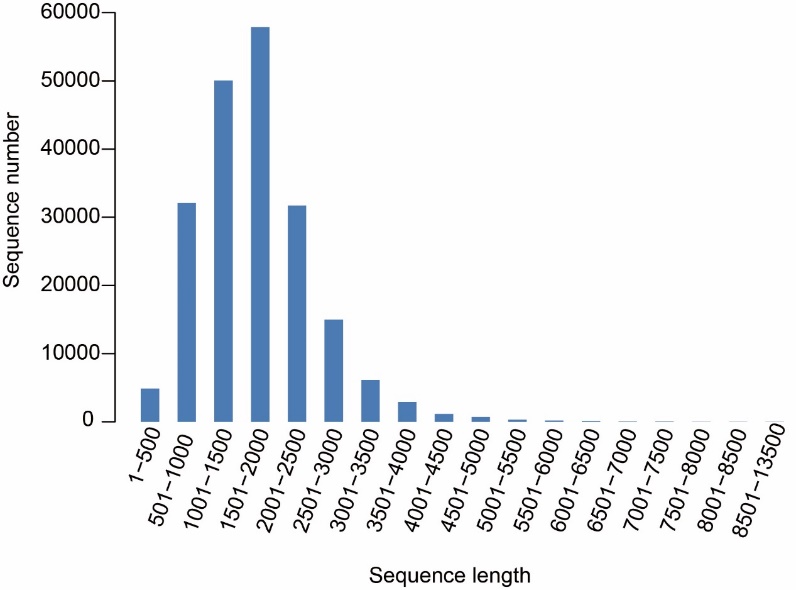


Fig. S3. Full length non-chimeric (FLNC) reads distribution.

**
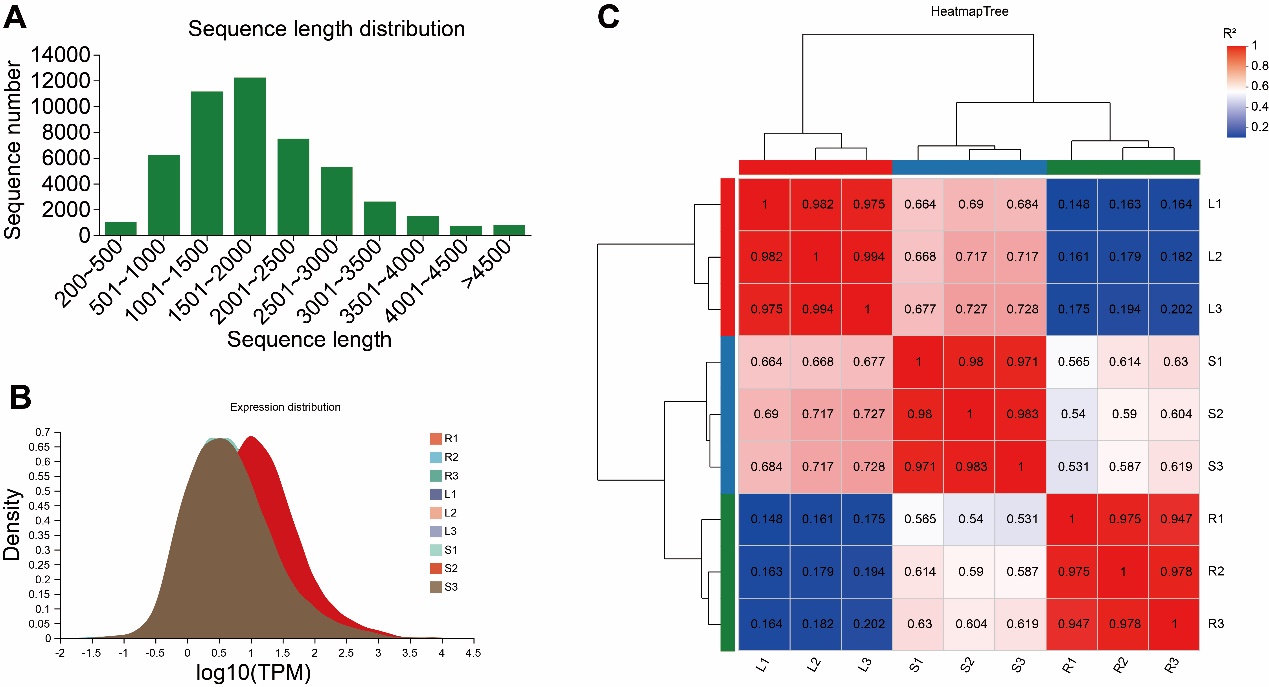
**

Fig. S4. De Novo Assembly and Expression Analysis of the Transcriptome

A. Assemble and obtain length statistics of all transcripts. B. FPKM density distribution of each sample. C. Correlation heatmap between samples.

**
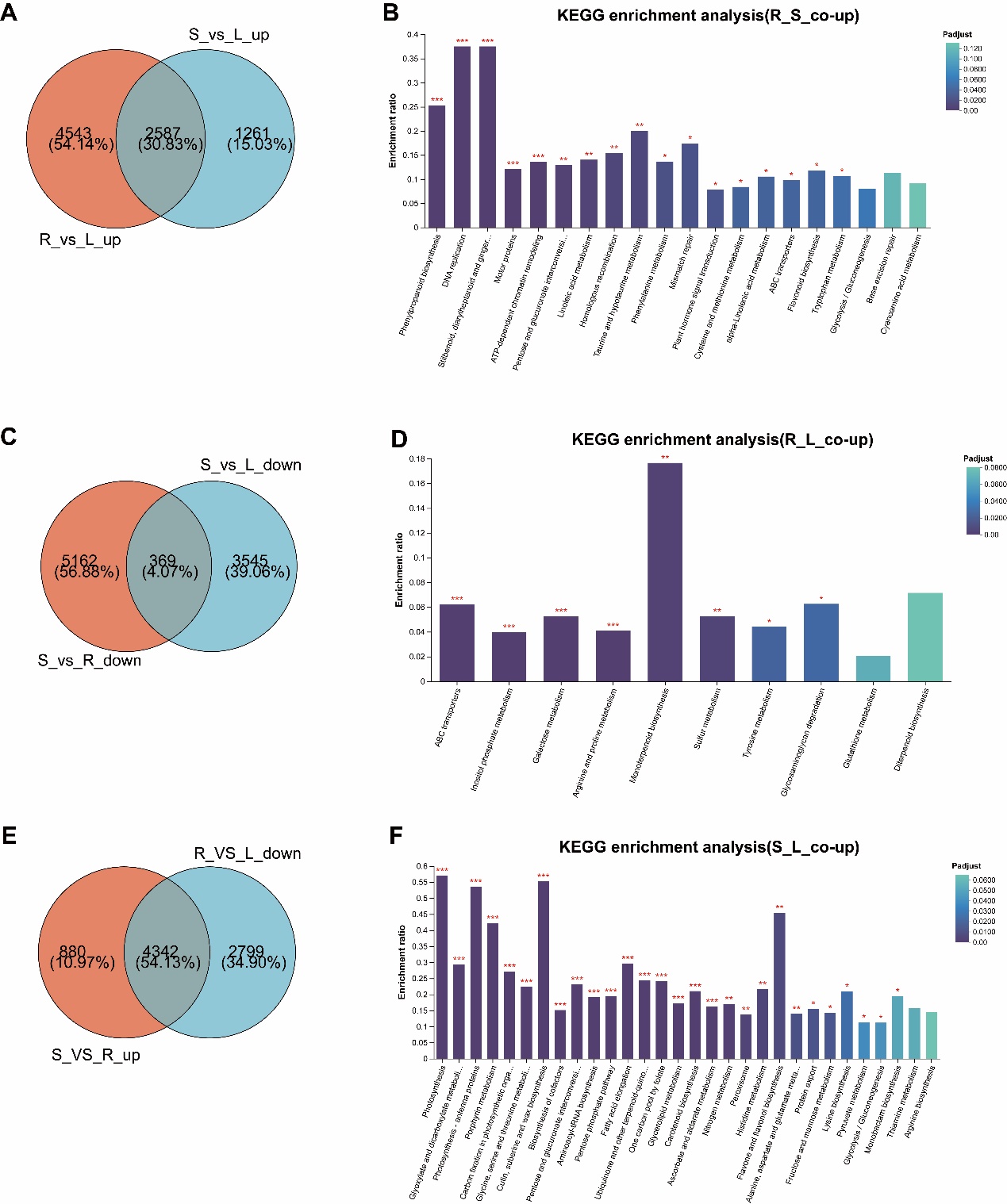
**

Fig. S5 Venn and KEGG analyzed the co-upregulated genes between the two tissues of Fenugreek (root, stem, leaf)

(A, C, E) Venn diagrams illustrating tissue-specific gene expression. The circles in different colors correspond to separate gene sets, with numerical values within the circles representing the counts of shared and unique genes across these sets.

(B, D, F) KEGG pathway analysis of tissue-specific expression genes, with the horizontal axis representing the KEGG pathways and the vertical axis representing the Rich factor. An increased Rich factor indicates a higher level of enrichment. The histogram's color gradient reflects the significance of enrichment, with Padjust < 0.001 indicated by ***, Padjust < 0.01 by **, and Padjust < 0.05 by *.


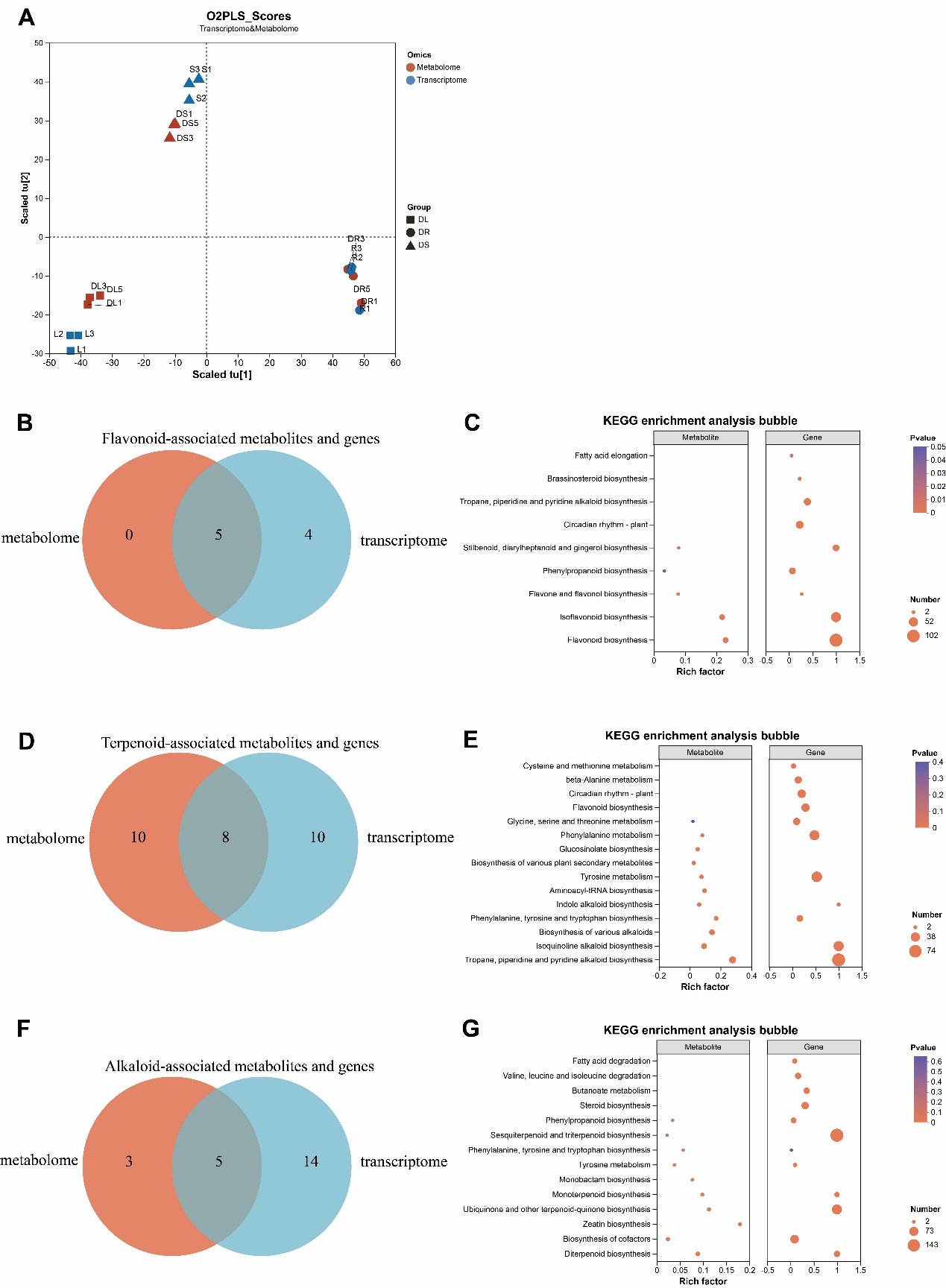


Fig. S6. KEGG Enrichment Analysis of Differential Gene and Metabolic Sets

(A) O2PLS integration of transcriptomic and metabolomic datasets. Axes represent joint projection scores. (B) Venn diagram of flavonoid-related KEGG pathways enriched by metabolites and genes. (C) Integrated map of KEGG pathways enriched by flavonoid-associated metabolites and genes. (D) Venn diagram of terpenoid-related KEGG pathways enriched by metabolites and genes. (E) Integrated map of KEGG pathways enriched by terpenoid-associated metabolites and genes. (F) Venn diagram of alkaloid-related KEGG pathways enriched by metabolites and genes. (G) Integrated map of KEGG pathways enriched by alkaloid-associated metabolites and genes.


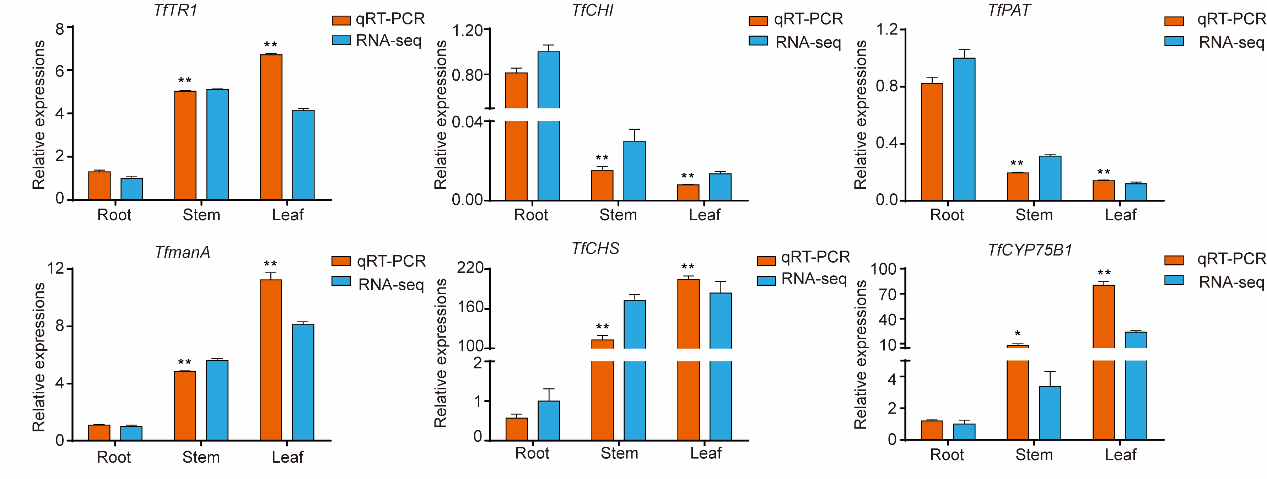


Fig. S7. Validation of RNA-seq results by qRT-PCR

The mRNA levels of the genes were quantified by qRT-PCR using *TfActin7* as the internal reference. The blue bars represent the gene expression levels determined by RNA-seq, while the orange bars represent the gene expression levels measured by qRT-PCR. Data are presented as mean ± standard deviation, with each sample comprising three independent biological replicates. **P* < 0.01, ***P* < 0.01 indicate statistically significant differences.


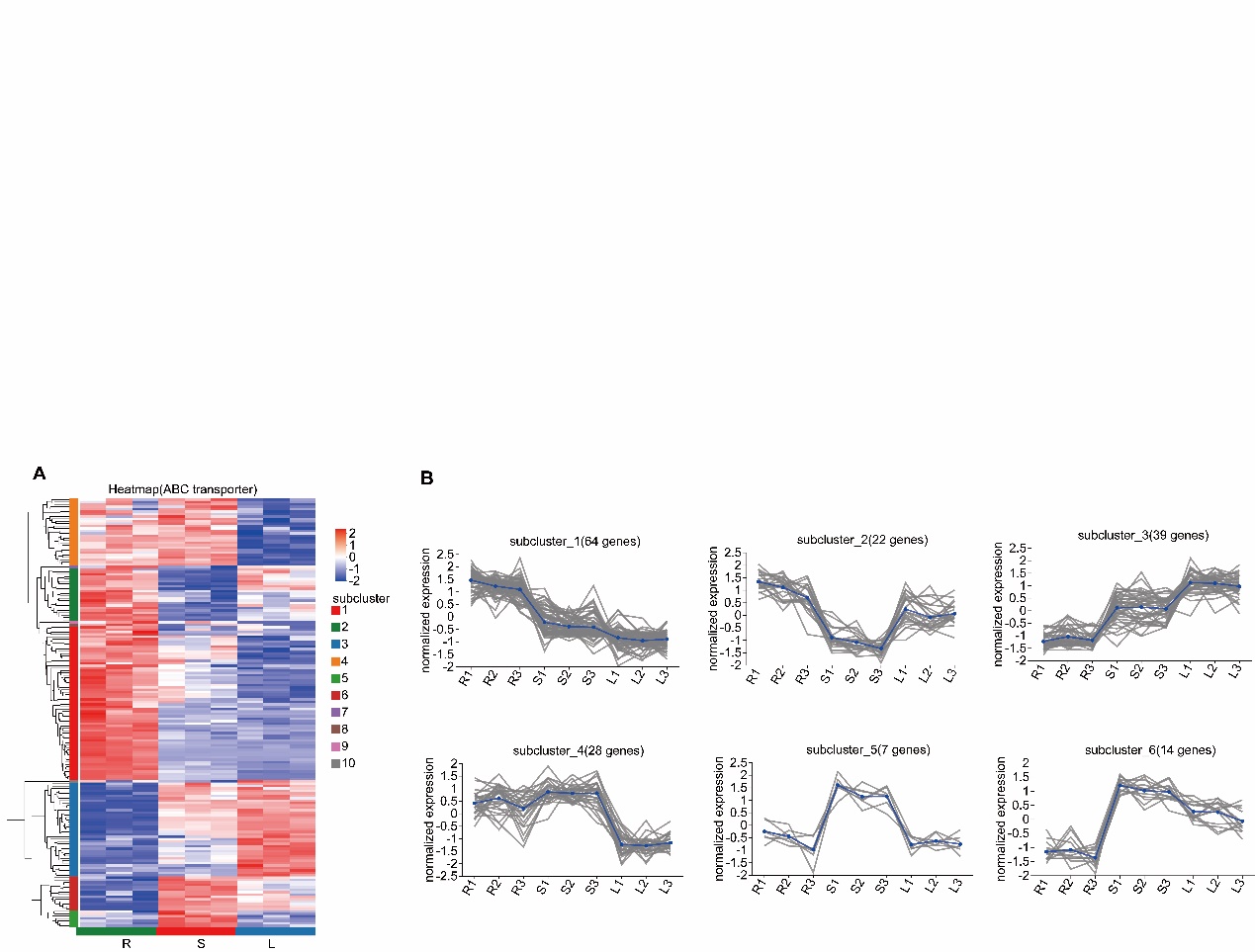


Fig. S8. Analysis of differentially expressed ABC transporter genes in tissues

A. Heatmap of hierarchical clustering analysis of ABC transporter genes; B. Statistical analysis of differentially expressed gene clusters.


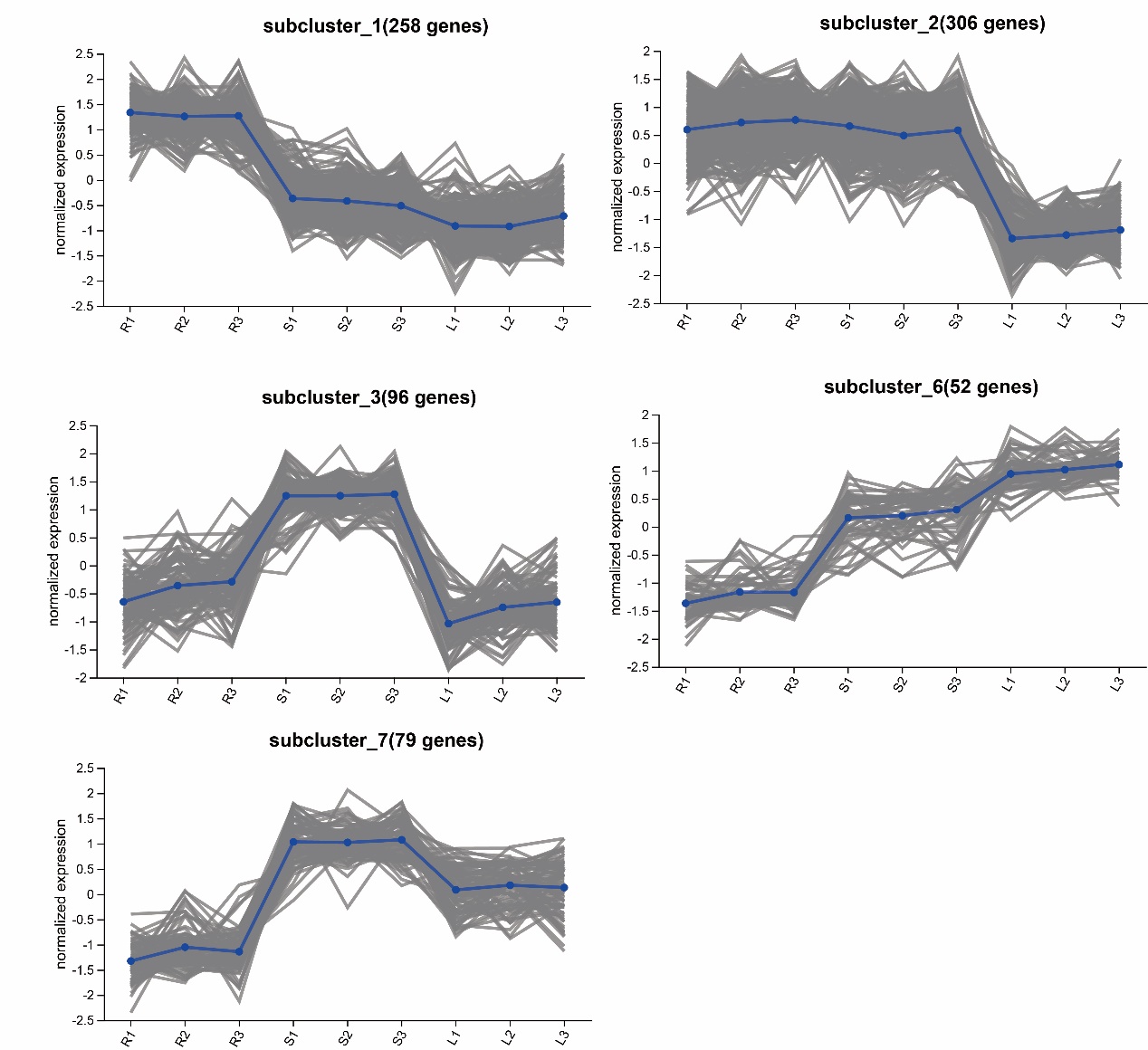


Fig. S9. Subclustering Trend of *TF* Sets

The horizontal axis represents the samples, and the vertical axis indicates the expression level of transcripts in each sample. Each gray line illustrates the expression trend of an individual transcript. The blue line represents the fitted trend of the mean expression value for all transcripts within the subcluster.
